# Spin‐Orbit Angular Momentum Conversion in Coupled Nonparaxial Bessel Acoustic Vortices

**DOI:** 10.1002/advs.202505631

**Published:** 2025-06-10

**Authors:** Di‐Chao Chen, Qian Mo, Da‐Jian Wu, Xing‐Feng Zhu, Ying Cheng, Xiao‐Jun Liu

**Affiliations:** ^1^ Institute of Acoustics School of Physics and Technology Nanjing Normal University Nanjing 210023 China; ^2^ MOE Key Laboratory of Modern Acoustics School of Physics Nanjing University Nanjing 210093 China

**Keywords:** acoustic flat structure, bessel acoustic vortex, spin‐orbit angular momentum conversion

## Abstract

Acoustic waves as longitudinal waves are recently demonstrated to have both spin and orbital angular momentums (AMs). The separation of orbital and spin components is proposed as the most current idea. However, it remains extremely difficult to develop studies sensitive to both types of AM using direct wave field measurements. Here, theoretical explanation and experimental proof of the acoustic spin‐orbit AM conversion are reported. A formulaic description of spin‐orbit AM conversion in coupled fields of two nonparaxial Bessel acoustic vortices is given by a thorough theoretical derivation. Experimental results of wave field measurements provide compelling evidence for spin‐orbit AM conversion via passive artificial structures. Based on this conversion, contactless high‐resolution manipulation of micro‐objects is further achieved, as demonstrated by the controlled rotation of Rayleigh particles and their translation along a circular trajectory. This work provides deeper insights into the AM physics of classical waves and has significant implications for acoustic sensing, acoustic imaging, and particle manipulation based on acoustic radiation force or torque.

## Graphical abstract

This study gives a formulaic description of the spin‐orbit angular momentum (AM) conversion in coupled fields of two nonparaxial Bessel acoustic vortices. Experimental results of wave field measurements provide compelling evidence for spin‐orbit AM conversion via passive artificial structures. Based on this conversion, this study has achieved contactless high‐resolution manipulation of microobjects.

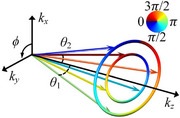

## 1 Introduction

Precise acoustic contactless object manipulation has shown great potential in engineering, biological, and chemical research, for example, handling delicate biological particles (e.g., exosomes and cells),^[^ ^1^, ^2^, ^3^ ^]^ transporting droplets of hazardous reagents, controlling the self‐assembly of colloidal materials, and arranging nanomaterials for the production of composites.^[^ ^4^, ^5^, ^6^ ^]^ Compared to other contactless manipulation methods, acoustic tweezers are biocompatible, can penetrate barriers (e.g., tissue, glass, Petri dishes, etc.), and can manipulate objects of different dimensions and material properties (e.g., soft, hard, gas, and liquid samples).^[^ ^7^, ^8^, ^9^ ^]^ Despite significant advances in acoustic contactless object manipulation mechanisms, achieving rotation of microobjects remains challenging.^[^ ^10^ ^]^ The capacity to rotate objects endows a novel degree of control for micro‐objects and has significant applications in micromachines, such as acoustically driven microrobots.^[^ ^11^, ^12^, ^13^ ^]^ Moreover, this capacity is imperative for 3D localization of biological samples (e.g., cells, spheres, embryos, and zebrafish larvae), which can be observed from multiple directions by rotation. Furthermore, the combination of particle manipulation with object rotation holds considerable promise for the bottom‐up assembly of small objects, where the orientation and position of each object can be meticulously controlled.^[^ ^14^ ^]^

Spin angular momentum (AM) inspires fundamental insights into topological problems, providing additional degrees of freedom for controlling wave propagation and wave‐matter interactions, with practical implications for manipulation devices.^[^ ^15^, ^16^, ^17^, ^18^, ^19^ ^]^ The AM of a transverse wave, such as an electromagnetic wave, can be separated into two parts: a spin component that is associated with the circular transverse polarization of the electric field^[^ ^20^, ^21^, ^22^ ^]^ and an orbital component that is related to the spatial distribution of the phase.^[^ ^23^, ^24^, ^25^ ^]^ In contrast, unlike transverse waves, which are circularly polarized, acoustic waves are longitudinal waves and cannot have spin AM due to their curl‐free characteristics.^[^ ^26^ ^]^ More recently, both theoretical and experimental evidence have shown the existence of finite spin AM densities after treating acoustics as vectorial velocity fields exclusively.^[^ ^27^, ^28^, ^29^, ^30^, ^31^, ^32^, ^33^, ^34^ ^]^ Toftul *et al.* further revealed the relationship between acoustic spin AM density and torque, providing a fundamental explanation for the use of acoustic spin AM to manipulate particle rotation.^[^ ^35^ ^]^ Nevertheless, the spin AM distribution of the vortex acoustic beam is constrained by the topological charge (TC), and the spatial integral is always equal to zero, resulting in the absence of spin degrees of freedom.

Spin‐orbit interactions^[^ ^36^, ^37^, ^38^, ^39^, ^40^ ^]^ between an isolated droplet^[^ ^41^ ^]^ and an evanescent wave propagating at the interface of two immiscible fluids have been demonstrated recently, with phases cycling around the singularity indicating a conversion of acoustic spin AM to orbital AM. However, the converted orbital AM must be obtained by measuring the acoustic field inside the isolated droplet, which implies that effective spin‐orbit conversion is impossible in acoustics without considering acoustic interface interactions. Thus, differentiating between the two forms of AM experimentally using direct wave field measurements remains a significant challenge. Recent proposals also have presented mechanistic evidence for the acoustic spin AM transmission to matter, as well as the separation of acoustic orbital and spin components.^[^ ^31^, ^35^ ^]^ However, the acoustic spin‐orbit AM conversion was rarely reported. How to use conversion to modulate acoustic spin AM and realize contactless high‐resolution complex manipulation of micro‐objects is an urgent problem at present.

In this work, the coupling between two nonparaxial Bessel acoustic vortices (BAVs) is proposed to achieve the acoustic spin‐orbit AM conversion. In general, the superposition of two vortices with opposite TCs leads to the destruction of the spatial distribution of the individual vortex phases and hence the disappearance of the orbital AM of the acoustic field.^[^ ^42^, ^43^ ^]^ If the BAVs have different polar angles, a conversion between acoustic spin and orbital AMs will occur during their interaction, resulting in the reappearance of the vanishing orbital AM. We have developed a formulaic description of this spin‐orbit AM conversion in the coupled two BAVs. An acoustic flat structure (AFS) carved with two sets of circular holes is further designed to demonstrate the acoustic spin‐orbit AM conversion. Both simulations and direct wave field measurements validate the theoretical prediction. Finally, we investigate the mechanical effects of the coupled acoustic fields on particles placed in them and perform particle complex manipulation experiments.

## 2 Spin‐Orbit Angular Momentum Conversion

**Figure 1:**
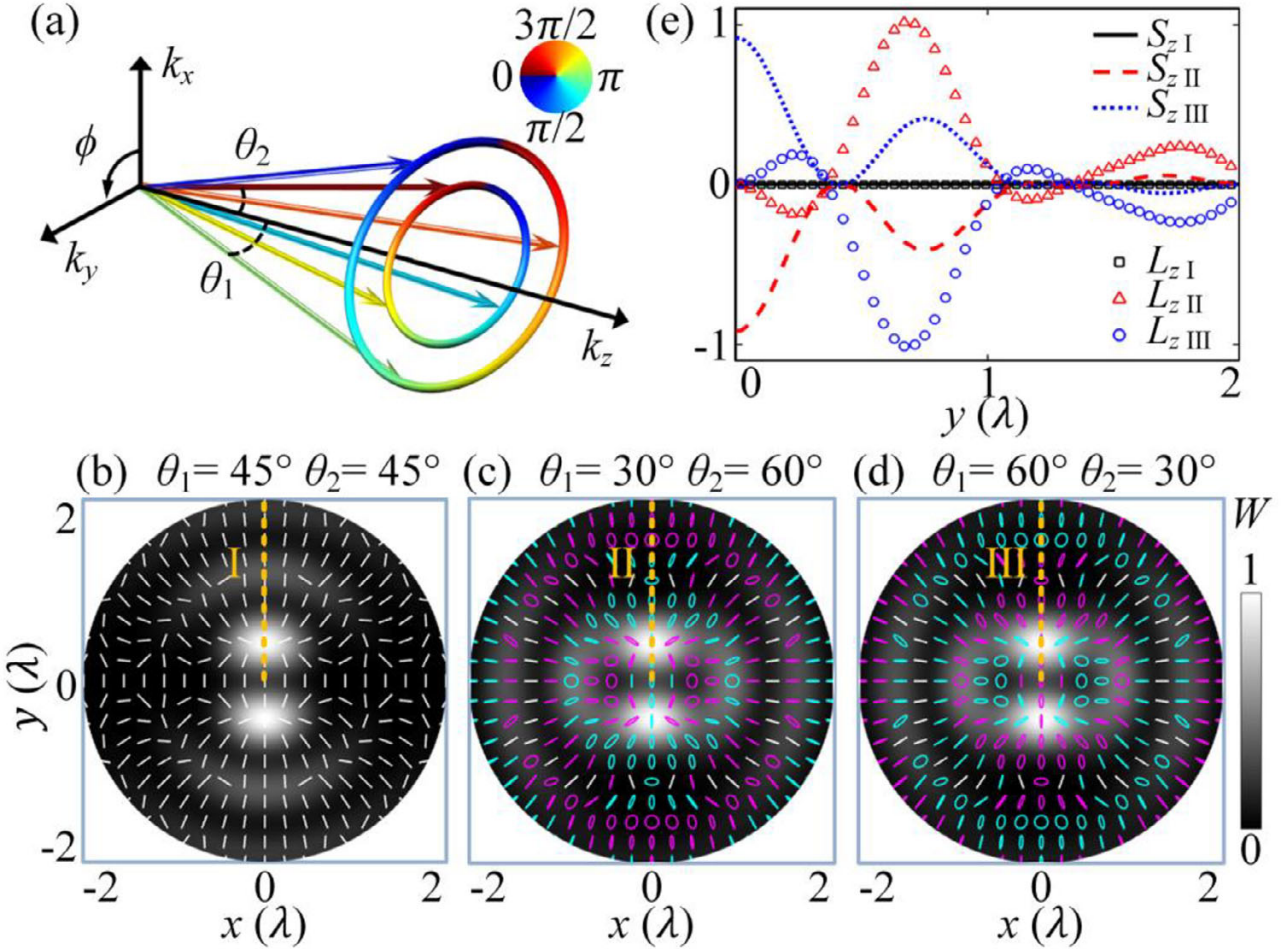
a) Schematics of the interaction of two nonparaxial Bessel acoustic vortices (BAVs) with opposite topological charges of *l*_1_ = 1 and *l*_2_ = −1 and polar angles of *θ*_1_ and *θ*_2_. Distributions of the energy‐density and polarization ellipses of the velocity field (*v_x_, v_y_*) of the coupled BAVs with b) (*θ*_1_ = 45° and *θ*_2_ = 45°), c) (*θ*_1_ = 30° and *θ*_2_ = 60°), and d) (*θ*_1_ = 60° and *θ*_2_ = 30°) in the transverse section. e) Spin and orbital AMs on the lines “I”, “II”, and “III” in (b), (c), and (d).

Consider the acoustic spin‐orbit AM conversion under BAV interaction. A higher‐order BAV propagating along the z‐axis can be represented as a superposition of plane waves of the same frequency and wave vector uniformly distributed on a circle with a fixed polar angle (*θ*).^[^ ^44^, ^45^, ^46^ ^]^ The phases of these plane waves are dependent on the azimuthal angle (*ϕ*) in *k*‐space, and as a consequence, they are capable of generating spiral phases and orbital AM in real space. We superimpose two nonparaxial higher order BAVs with opposite TCs as in **Figure** **1**a. The superimposed pressure field is described as

(1) $$\begin{array}{ccc} P=P_{1}+P_{2} & = & AJ_{l_{1}}\left(k_{r_{1}}r\right)\exp\left(il_{1}\varphi +ik_{z_{1}}z\right)\\ & & +\;BJ_{l_{2}}\;\left(k_{r_{2}}r\right)\exp\left(il_{2}\varphi +ik_{z_{2}}z\right) \end{array}$$

where *A* and *B* are the constant amplitudes of the two acoustic beams, *l*_1_ and *l*_2_ are the TCs of the two beams, $k_{z_{\;1}}=k\cos\theta_{1}$ and $k_{z_{\;2}}=k\cos\theta_{2}$ are the axial wave numbers of the two beams, $k_{r_{\;1}}=k\sin\theta_{1}$ and $k_{r_{\;2}}=k\sin\theta_{2}$ are the radial wave numbers of the two beams, and (*r*, *φ*, *z*) are the cylindrical coordinates in real space. The vector velocity part of the pressure field can be obtained from $\nabla P=i\rho \omega \;\vec{v}$, where *ω* is the angular frequency of the acoustic wave and *ρ* is the mass density. Substituting into Equation (1) yields the radial, angular, and axial velocity components

(2) $$\left\{\begin{array}{c} v_{r}=-i\frac{Ak_{r_{1}}}{2\rho \omega }\left[J_{l_{1}-1}\left(k_{r_{\;1}}r\right)-J_{l_{1}+1}\left(k_{r_{1}}r\right)\right]exp\left(il_{1}\varphi +ik_{z_{1}}z\right)\\ -i\frac{Bk_{r_{2}}}{2\rho \omega }\left[J_{l_{2}-1}\left(k_{r_{2}}r\right)-J_{l_{2}+1}\left(k_{r_{2}}r\right)\right]exp\left(il_{2}\varphi +ik_{z_{2}}z\right),\\ v_{\varphi }=\frac{Ak_{r_{\;1}}}{2\rho \omega }\left[J_{l_{\;1}-1}\left(k_{r_{\;1}}r\right)+J_{l_{1}+1}\left(k_{r_{\;1}}r\right)\right]exp\left(il_{1}\varphi +ik_{z_{1}}z\right)\\ +\frac{Bk_{r_{2}}}{2\rho \omega }\left[J_{l_{2}-1}\left(k_{r_{2}}r\right)+J_{l_{2}+1}\left(k_{r_{2}}r\right)\right]exp\left(il_{2}\varphi +ik_{z_{2}}z\right),\\ v_{z}=\frac{Ak_{z_{\;1}}}{\rho \omega }J_{l_{1}}\left(k_{r_{1}}r\right)\exp\left(il_{1}\varphi +ik_{z_{1}}z\right)+\frac{Bk_{z_{2}}}{\rho \omega }J_{l_{2}}\left(k_{r_{2}}r\right)\exp\left(il_{2}\varphi +ik_{z_{2}}z\right) \end{array}\right.$$

Substituting the vector velocity part of the superimposed acoustic field $W=\frac{1}{4}\left(\beta {\left|P\;\right|}^{2}+\rho {|\vec{v}\;|}^{2}\right)$ and using the dispersion relation $\omega^{2}=k^{2}c^{2}\equiv \frac{k^{2}}{\rho \beta }$, the energy density distribution can be obtained

(3) $$\begin{array}{ccc} W & = & \frac{\beta A^{2}}{8}\left\{2\left(1+\cos^{2}\theta_{1}\right)J_{l_{\;1}}^{2}\left(k_{r_{1}}r\right)\right.\\ & & \left.+\sin^{2}\theta_{1}\left[J_{l_{1}-1}^{2}\left(k_{r_{1}}r\right)+J_{l_{1}+1}^{2}\left(k_{r_{1}}r\right)\right]\right\}\\ & & +\frac{\beta B^{2}}{8}\left\{2\left(1+\cos^{2}\theta_{2}\right)J_{l_{2}}^{2}\left(k_{r_{2}}r\right)\right.\\ & & \left.+\;\sin^{2}\theta_{2}\left[J_{l_{2}-1}^{2}\left(k_{r_{2}}r\right)+J_{l_{\;2}+1}^{2}\left(k_{r_{2}}r\right)\right]\right\}\\ & & +\frac{\beta AB}{8}\left\{2\left(1+\cos\theta_{1}\cos\theta_{2}\right)J_{l_{1}}\left(k_{r_{1}}r\right)J_{l_{2}}\left(k_{r_{\;2}}r\right)\right.\\ & & \left.+\;\sin\theta_{1}\sin\theta_{2}\left[J_{l_{1}-1}\left(k_{r_{1}}r\right)J_{l_{2}-1}\left(k_{r_{2}}r\right)\right.\right.\\ & & \left.\left.+\;J_{l_{1}+1}\left(k_{r_{1}}r\right)J_{l_{2}+1}\left(k_{r_{2}}r\right)\right]\right\}\\ & & \left\{\exp\left[i\left(l_{1}-l_{2}\right)\varphi +i\left(k_{z_{1}}-k_{z_{2}}\right)z\right]\right.\\ & & \left.+\exp\left[-i\left(l_{1}-l_{2}\right)\varphi -i\left(k_{z_{1}}-k_{z_{2}}\right)z\right]\right\} \end{array}$$

where *β* is the inverse of the bulk modulus. Setting *A* = *B* = 1 and the TCs of the two BAVs are *l*_1_ = 1 and *l*_2_ = −1, respectively. The energy density distributions with different combinations of pole angles are shown in greyscale background Figure 1b–d. We first note that the superposition of two beams can produce an energy density distribution with a double strong point. As the polar angle of the two BAVs undergoes a change, the energy density distribution undergoes a slight alteration, while the double strong point remains a constant presence. As with electromagnetic waves, the polarization ellipse in the transverse (*x*, *y*) plane is related to the *z*‐component of the spin AM density. The transverse polarization distributions of the superimposed beam velocity fields (*v_x_*, *v_y_*) are shown in Figure 1b–d. It can be observed that when the two BAVs have the same polar angle, the superimposed field exhibits purely linear radial polarization [Figure 1b]. In the event that the two BAVs possess disparate polar angles, the superimposed Bessel sound field exhibits a variable polarization. Comparing Figure 1c,d, the acoustic field will have an opposite polarization distribution when the polar angles of the two BAVs are flipped. To provide a more illustrative account of the conversion of acoustic spin‐orbit AM in the presence of BAV interactions, we calculate the spin and orbital AMs of the superimposed acoustic field. Substituting Equation (2) $\vec{S}=\frac{\rho }{2\omega }Im\;\left({\vec{v}}^{*}\times \vec{v}\right)$, we obtain the spin AM of the superimposed sound field. The orbital AM can be obtained from $\vec{L}=\vec{r}\times \vec{p}$. Here, $\vec{p}=\frac{\vec{\Pi }}{c^{2}}-\frac{1}{4}\nabla \times \vec{S}$ is the canonical momentum density, and $\vec{\Pi }=\frac{1}{2}Re\left(P^{*}\vec{v}\right)$ is the energy flux density. Expressions for the spin AM and orbital AM of the coupled acoustic field after superposition can be found in Section 1 of Supporting Information. We show the axial components of spin and orbital AMs along the dashed lines “I”, “II” and “III” [marked in Figure 1b–d] in Figure 1e. It is evident that the spin and orbital AMs of the two BAVs with identical polar angles are both zero. Altering the polar angles of the two BAVs leads to corresponding changes in the spin and orbital AMs of the superimposed acoustic field. Incorporating the spin AM into the orbital AM expression yields the general conversion formula for the axial spin and orbital AMs after the superposition of two BAVs with opposite TCs as

(4) $$L_{z}=\frac{\left(l_{1}W_{1}-l_{1}W_{2}\right)}{\omega }-\frac{S_{z}}{2}$$

Equation (4) is the central result of this article. Here,

(5) $$\left\{\begin{array}{c} W_{1}=\frac{\beta }{8}\left\{2\left(1+\cos^{2}\theta_{1}\right)J_{l_{\;1}}^{2}\left(k_{r_{\;1}}r\right)+\sin^{2}\theta_{1}\left[J_{l_{\;1}-1}^{2}\left(k_{r_{\;1}}r\right)+J_{l_{\;1}+1}^{2}\left(k_{r_{\;1}}r\right)\right]\right\},\\ W_{2}=\frac{\beta }{8}\left\{2\left(1+\cos^{2}\theta_{2}\right)J_{l_{\;2}}^{2}\left(k_{r_{\;2}}r\right)+\sin^{2}\theta_{2}\left[J_{l_{\;2}-1}^{2}\left(k_{r_{\;2}}r\right)+J_{l_{\;2}+1}^{2}\left(k_{r_{\;2}}r\right)\right]\right\}, \end{array}\right.$$

are the time‐averaged energy densities of the two BAVs, respectively. It is shown that the spin and orbital AMs of the superimposed acoustic field are only related to the energy densities (*W*_1_ and *W*_2_) of the single beams, but not to the energy density *W* of the superimposed acoustic field. In general, the acoustic field after the superposition of vortices of opposite TCs will no longer have orbital AM. Nevertheless, Equation (4) indicates that the interaction of BAVs with different polar angles will be accompanied by an acoustic spin‐orbit AM conversion. This allows for the manipulation of the spin AM of the superimposed acoustic field and causes vanishing orbital AM to reappear.

## 3 Design Theory of Acoustic Flat Structure

**Figure 2:**
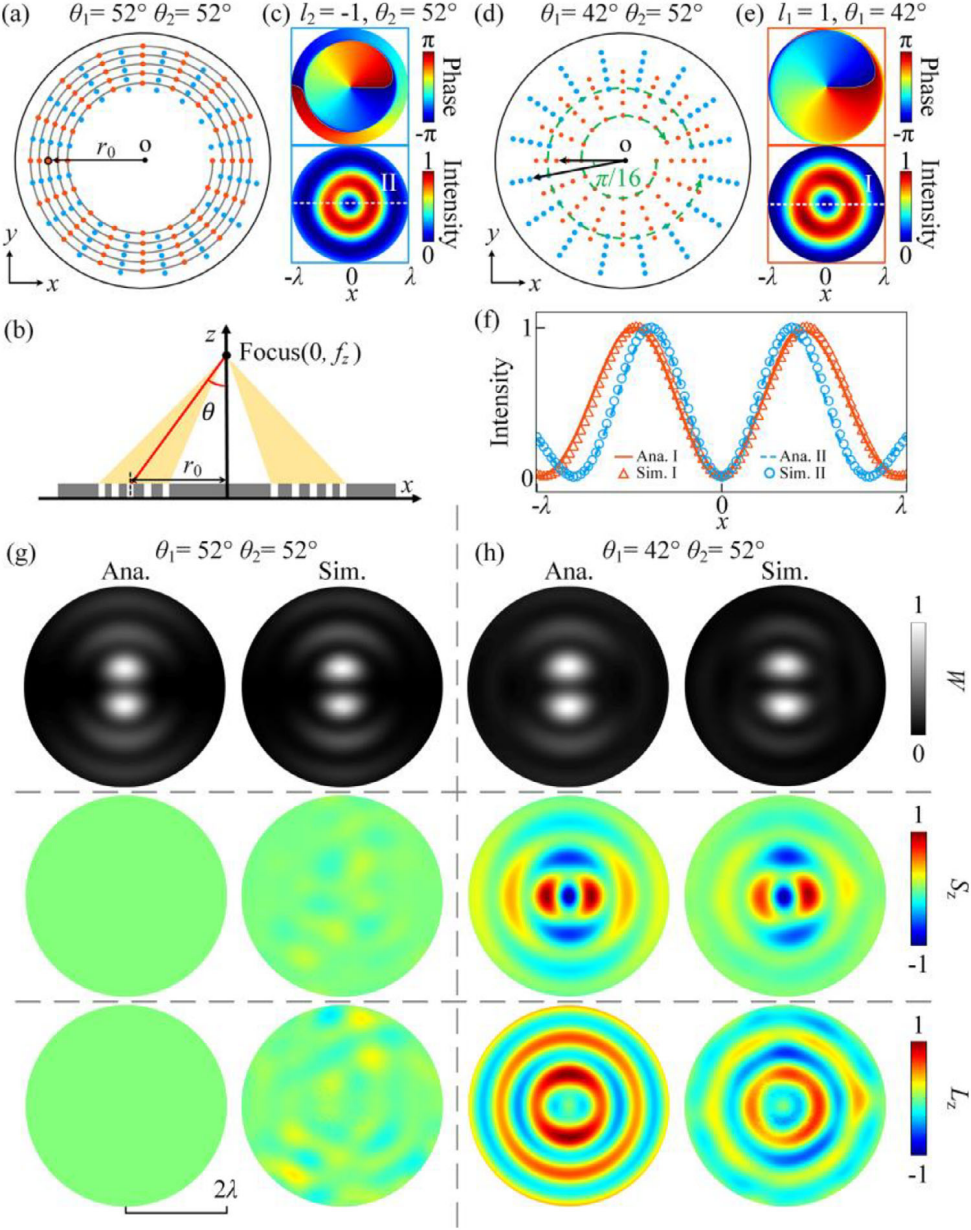
a) Schematic of acoustic flat structure (AFS) carved with two sets of circular holes for generating two focused BAVs with (*l*_1_ = 1 and *θ*_1_ = 52°) (orange) and (*l*_2_ = −1 and *θ*_2_ = 52°) (blue). Here, *r*_0_ is the initial radius of the helix and the diameter of the circular hole is fixed at *d*_1_ = *d*_2_ = 14 mm. b) Schematic diagram of the generation of a BAV with a polar angle *θ* after a planar acoustic wave passes through an AFS with 5‐turn helical circular holes. c) Phase and intensity distributions of the acoustic fields on the focal plane after a planar acoustic wave passes through the blue circular holes in (a). d) Schematic of AFS carved with two sets of circular holes for generating two focused BAVs with (*l*_1_ = 1 and *θ*_1_ = 42°) and (*l*_2_ = −1 and *θ*_2_ = 52°). Here, the diameters of the circular holes are fixed at *d*_1_ = 10 mm and *d*_2_ = 14 mm, respectively. e) Phase and intensity distributions of the acoustic fields on the focal plane after a planar acoustic wave passes through the orange circular holes in (d). f) Acoustic intensity distributions on lines “I” and “II” in (c) and (d). Theoretical and simulated energy density, axial spin AMs density, and axial orbital AMs density distributions of the coupled acoustic field with g) (*θ*_1_ = 52° and *θ*_2_ = 52°) and h) (*θ*_1_ = 42° and *θ*_2_ = 52°) on the focal plane.

We plan to design an AFS to experimentally verify our theoretical predictions. Two sets of circular holes carved into the AFS are used to generate two focused BAVs with specific polar angles, as shown in **Figure** **2**a. The 80 orange circular holes are distributed in a uniform manner on a 5‐turn helix, and the orbit of the 5‐turn helix can be represented in polar coordinates as^[^ ^34^ ^]^

(6) $$r^{2}={\left[\sqrt{{r_{0}}^{2}+f_{z}^{2}}+\frac{\alpha \lambda }{2\pi }\right]}^{2}-f_{z}^{2},\;\;\;\left(-5\pi \leq \alpha \leq 5\pi \right)$$

where *f*_z_ is the focal length, *λ* is the wavelength, *α* is the azimuth of the helix, and the constant *r*_0_ is defined as the initial radius. The distance from any point on the helix to the focus is $L=\sqrt{f_{z}^{2}+r^{2}}$. Thus the phase difference between two neighboring points to the focus is $d\vartheta =\frac{2\pi }{\lambda }dL$. After integration, the one‐turn helix achieves a linear phase shift of 2π, satisfying the requirement that TC is 1. The acoustic field at the observation point (*ρ*, *φ*, *z*) can be calculated by^[^ ^47^ ^]^

(7) $$P\left(\rho ,\varphi ,z\right)=A_{0}\exp\left(ik_{z}z\right)\int_{-5\pi }^{5\pi }\frac{\exp\left[i{\vec{k}}_{r}\cdot \left(\rho {\hat{e}}_{\rho }-r{\hat{e}}_{r}\right)\right]}{R}d\alpha$$

where $R=\sqrt{{\left(\rho \cos\theta -r\cos\alpha \right)}^{2}+{\left(\rho \sin\varphi -r\sin\alpha \right)}^{2}+z^{2}}$, *A*_0_ is a real constant, *k_r_* and *k_z_* are the axial and radial wavenumbers, respectively. Consider the case $\sqrt{r_{0}^{2}+f_{z}^{2}}>>\alpha \lambda /2\pi$. When the observation point is in the vicinity of the focused vortex beam on the propagation axis, *R* can be approximated as $R\approx \sqrt{r_{0}^{2}+f_{z}^{2}}$. Thus, Equation (7) can be further approximated as

(8) $$\begin{array}{c} P\left(\rho ,\varphi ,z\right)\approx \frac{A_{0}}{\sqrt{r_{0}^{2}+f_{z}^{2}}}\exp\left(ik_{z}z\right)\exp\left(-ik_{r}r_{0}\right)\\ \times \int_{-5\pi }^{5\pi }\exp\left[i\rho k_{r}\cos\left(\varphi -\alpha \right)\right]\exp\left[-ik_{r}\left(r-r_{0}\right)\right]d\alpha , \end{array}$$

where $k_{z}=k\cos\theta \approx k\cdot f_{z}/\sqrt{r_{0}^{2}+f_{z}^{2}}$ and $k_{r}=\sqrt{k^{2}-k_{z}^{2}}$. The product of *k_r_* and (*r−r*_0_) is approximately equal to *α*. Then, using the Jacobi‐Anger expansion,^[^ ^47^ ^]^ Equation (8) can be further simplified as

(9) $$P\left(\rho ,\varphi ,z\right)\approx A\exp\left(i\varphi \right)\exp\left(ik_{z}z\right)J_{1}\left(k_{r}\rho \right)$$

where $A\approx \frac{A_{0}}{\sqrt{r_{0}^{2}+z^{2}}}10\pi \exp\left[-ik_{r}r_{0}-i\frac{\pi }{2}\right]$. Equation (9) demonstrates that 80 orange circular holes distributed along a 5‐turn helix can be employed to generate a BAV with *l* = 1. Furthermore, it is evident that the polar angle of the BAV can be modified through either the focal length (*f*_z_) or the initial radius (*r*_0_). Figure 2b shows the generation of a BAV with a *θ*‐polar angle after a planar acoustic wave passes through an AFS with a 5‐turn helical circular hole. Another set of 80 blue circular holes is located on a counter‐rotating 5‐turn helical trajectory, and they can produce a BAV with opposite TC in the focal plane. In the following discussion, we denote the diameter of the orange circular hole as *d*_1_ and the initial radius of the helix in which it is located as *r*_o_, and the diameter of the blue circular hole as *d*_2_ and the initial radius of the helix in which it is located as *r*_b_. Here, two sets of discrete circular holes that do not intersect each other are chosen so that the BAV produced by each set of circular holes can be manipulated independently. The amplitude of the beam is adjusted by varying the size of the circular holes (*d*_1_ and *d*_2_) to ensure that the amplitudes of the two BAVs produced are consistent with the theory. The two sets of circular holes are arranged alternately and the crossing angle of the two sets of circular holes is fixed at π/16. Figure 2a shows the AFS that produces *l*_1_ = 1, polar angle *θ*_1_ = 52° and *l*_2_ = −1, polar angle *θ*_2_ = 52° BAVs. Here *d*_1_ = *d*_2_ = 0.6*λ*, *r*_o_ = *r*_b_ = 13*λ* and *f*_z_ = 10*λ * are set. Full‐wave simulations are conducted based on the finite‐element method, utilizing the COMSOL Multiphysics software, to verify the design further. The background setting is air with a mass density of 1.21 kg·m^−3^ and a speed of sound of 343.2 m·s^−1^. The operational frequency is fixed at 15 kHz (*λ* = ≈2.29 cm), *f*_z_ is fixed at 22.9 cm (10*λ*) and the AFS is assumed to be acoustically rigid. We first verified the BAV generated by the blue circular hole in Figure 2a. The diameter of the blue circular hole is *d*_2_ = 14 mm and *r*_b_ = 29.6 cm. The first row of Figure 2c depicts the phase distribution at the focal plane of a planar acoustic wave after passing through the blue circular holes. It can be observed that it has a screw phase dislocation with a singularity at the center and the TC equal to −1. In this study, we set the counterclockwise phase increase direction as the positive direction of rotation. The normalized simulated acoustic intensity distributions in the second row show the null at the axis characteristic of the first‐order BAV. Without changing the focal length, the polar angle of the generated BAV can be adjusted by the initial radius *r*_o_ or *r*_b_. According to Equation (8), the polar angles and amplitudes of the generated BAVs are both related to the initial radius of the helix where the circular holes are located. To satisfy the requirement for the polar angle, it is necessary to alter the initial radius of the helix. However, this adjustment also leads to a change in the amplitude of the generated BAV. Therefore, we need to ensure that the focused BAV produced by the orange holes has the same amplitude as that generated by the blue holes by changing the size of the orange hole. In Figure 2d, the diameter of the orange circular hole is replaced by *d*_1_ = 10 mm and the value of *r*_o_ is replaced with 20.7 cm, thereby modifying the polar angle of the BAV generated by the orange circular holes to *θ*_1_ = 42°. Figure 2e shows the phase and normalized simulated acoustic intensity distributions at the focal plane of a planar acoustic wave after passing through these orange circular holes. It has the opposite TC compared to the phase in Figure 2c. To show the generated BAVs more clearly, we compare the simulation results with the theory in Figure 2f. The orange solid line and the blue dashed line are the theoretical radial intensity distributions of the first‐order BAVs with polar angle *θ*_1_ = 42° and polar angle *θ*_2_ = 52°, respectively. The orange triangular symbols and blue circular symbols show the intensity contrast along lines “I” and “II” in Figure 2c,e, respectively. The simulation results (symbols) are in good agreement with the theory (curves), which shows that the polar angle and amplitude of the generated BAV can be adjusted by modifying the initial radius of the helical trajectory and the size of the circular hole (*d*_1_ and *d*_2_).

Figure 2g,h shows the energy density distributions, axial spin, and axial orbital AMs density distributions of the plane wave after passing through the AFSs of Figure 2a,d, respectively, and are compared with the theoretical results. It can be seen that they both have a double strong point energy density distribution. In the case where the polar angles of the two BAVs are identical, the resulting axial spin and axial orbital AMs densities are both found to be equal to zero. Conversely, a change in the polar angles of the two BAVs results in a conversion between spin and orbital AMs. The simulation results align with the theoretical analysis, demonstrating that the designed AFS is capable of converting spin‐orbit AM. Furthermore, the acoustic intensity, phase, and spin AM density distributions near the focusing plane and the frequency response characteristics of the spin‐orbit AM conversion are investigated (see Sections 2 and 3 in the Supporting Information). The findings demonstrate that spin‐orbit AM conversion remains highly effective when the observation plane is in proximity to the focusing plane or when the operating frequency fluctuates within the range of 8% (i.e., 13.8–16.2 kHz). We should note that the geometry of the holes on the AFS is set to be circular to facilitate sample fabrication, holes of other geometries can also be realized in the acoustic spin‐orbit AM conversion.

## 4 Direct Wave Field Measurement

**Figure 3:**
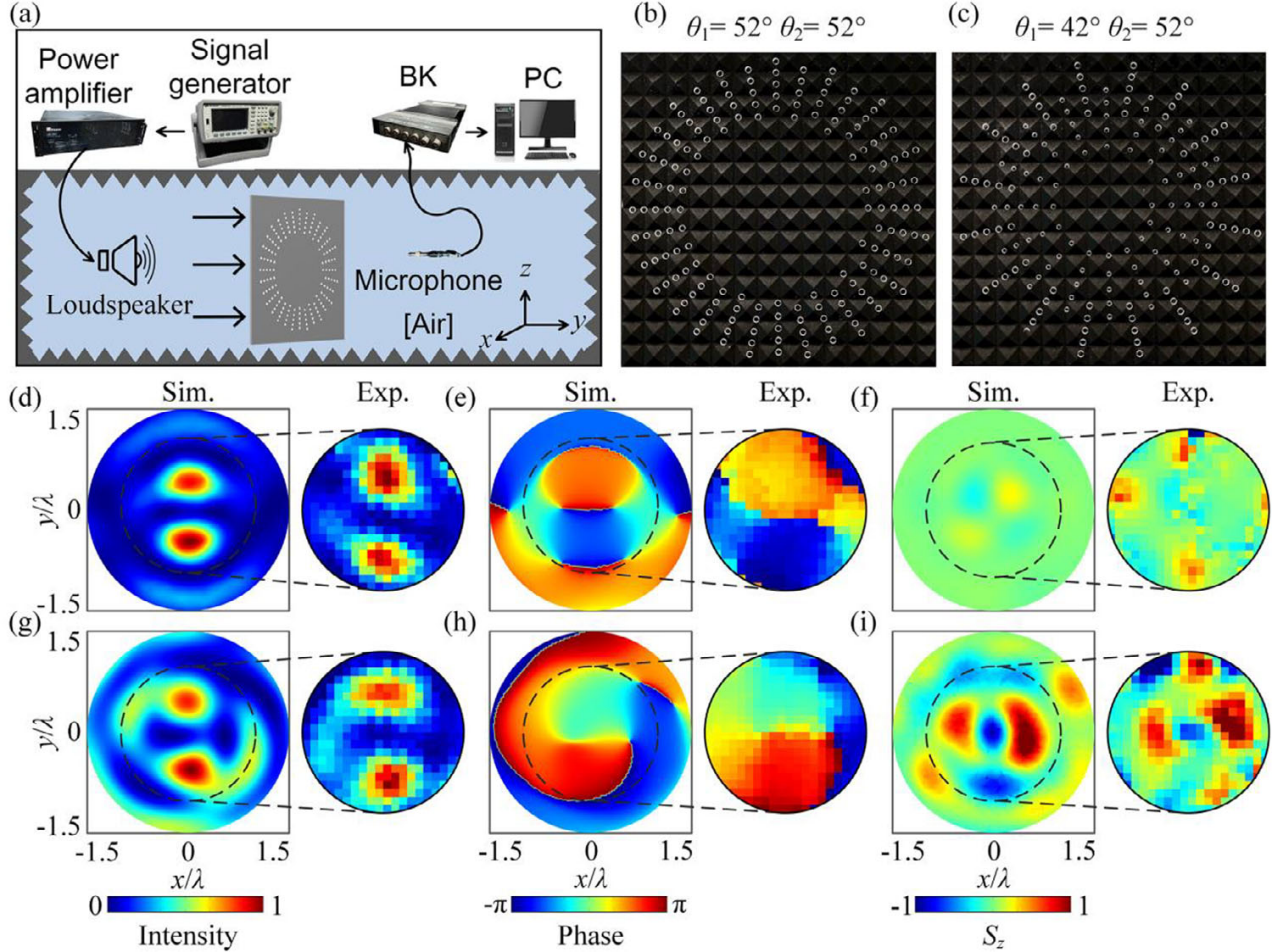
a) Experimental setup. Photographs of the AFS samples with b) (*θ*_1_ = 52° and *θ*_2_ = 52°) and c) (*θ*_1_ = 42° and *θ*_2_ = 52°). Simulated and measured acoustic intensity, phase, and spin AM density distributions of the coupled acoustic field on the focal plane after a quasi‐planar wave passes through the AFSs with d–f) (*θ*_1_ = 52° and *θ*_2_ = 52°) and g–i) (*θ*_1_ = 42° and *θ*_2_ = 52°).

We fabricated AFSs to verify acoustic spin‐orbit AM conversions under BAV interactions. The experimental system is constructed in an anechoic environment, as illustrated in **Figure** **3**a. The electrical signal from the signal generator is passed through a power amplifier and connected to a loudspeaker (Enpar Type‐PD2101). The loudspeaker is placed 1.4 m from the left side of the AFS to generate an incident continuous quasi‐plane wave with a center frequency of 15 kHz. A microphone (1/4 in., B&K Type‐4938‐A‐011) attached to a 3D stepper motor is placed on the right side of the AFS, and the sound field of the target area on the right side of the AFS is scanned point by point by this movable microphone with a 2.29‐mm (≈0.1 *λ*) step size. A multi‐analyzer system (B&K Type‐3160‐A‐042) is used to measure the time‐domain acoustic signals in the target area, and then the obtained acoustic signals are subjected to a Fourier transform to obtain the frequency spectrum of the signals. It is possible to reconstruct the acoustic intensity field from this data.

Figure 3b,c shows photographs of AFSs fabricated from polymethylmethacrylate, corresponding to the AFSs in Figure 2a,d, respectively. Previous studies were reported to show that polymethylmethacrylate plates can be considered acoustic hard boundaries in air.^[^ ^48^ ^]^ The geometric dimensions of the plates are 120 × 120 × 0.8 cm. Figure 3d–f and g–i shows the acoustic intensity, phase, and spin AM density distributions of the quasi‐planar wave after it passes through the artificially structured plate in Figure 3b,b, respectively. The normalized simulated and measured acoustic intensity distributions at 1.4 m from the AFS are shown in Figure 3d,g. It can be observed that both plates can produce acoustic intensity distributions with double high‐intensity lobes. Observing the phase distribution, we can notice the appearance of cycling around the phase singularity in Figure 3e compared to the discrete phase distribution in Figure 3h. The circulation of the phase of the wave around the singularity is a sign of the orbital AM transmitted to the fluid. Based on the acoustic intensity and phase we have computed the axial spin AM density distribution in Figure 3f,i. In agreement with theoretical predictions, when *r*_o_ and *r*_b_ are the same, the resulting axial spin AM density is 0 [Figure 3f]. When *r*_o_ and *r*_b_ are different, an axial spin AM density that is not 0 is produced [Figure 3i]. The measured results are in good agreement with the simulation results and also satisfy the general law of spin‐orbit AM conversion after the interaction of two BAVs in Equation (4). The discrepancy between the simulated and experimental results is primarily attributable to the generation of quasi‐plane waves by a single loudspeaker. Additionally, the measurements may be influenced by inaccuracies in the 3D stepper motor, sample fabrication errors, and unavoidable viscous effects. In light of the experimental outcomes, it is thought that the proposed artificial structure has the potential to realize the spin‐orbit AM conversion.

## 5 Applications in Particle Manipulation

The proposed AFSs can be directly used to produce coupled ultrasonic fields in water. Thus, we can investigate microparticle manipulation using them. The background medium is water with a mass density of *ρ*_0_ = 1000 kg·m^−3^ and a sound speed of *c*_0_ = 1500 m·s^−1^. To facilitate analysis, the AFS is regarded as acoustically rigid in water. The operational frequency and the focal length are fixed at 0.5 MHz (*λ* = 3 mm) and *f*_z_ = 30 mm (10*λ*), respectively.

**Figure 4:**
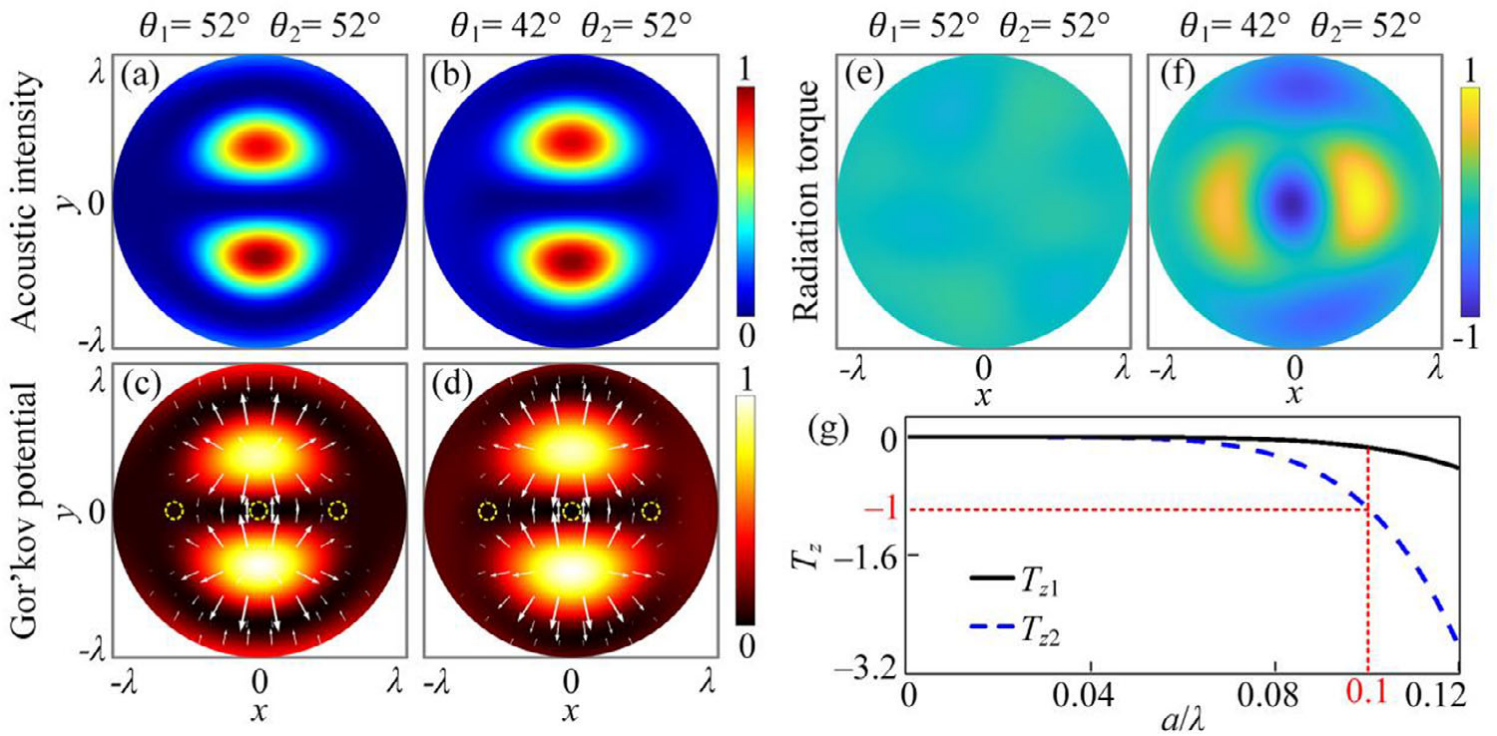
Normalized simulated acoustic intensity distributions of the coupled acoustic field on the focal plane after a plane wave passes through the AFSs with a) (*d*_1_ = *d*_2_ = 1.8 mm, *θ*_1_ = 52° and *θ*_2_ = 52°) and b) (*d*_1_ = 1.4 mm, *d*_2_ = 1.8 mm, *θ*_1_ = 42° and *θ*_2_ = 52°). The working frequency is set as 0.5 MHz. Gor'kov potential distributions of Rayleigh PS particles in the coupled acoustic fields generated by the AFSs with c) (*θ*_1_ = 52° and *θ*_2_ = 52°) and d) (*θ*_1_ = 42° and *θ*_2_ = 52°). The orientation and magnitude of the white arrows indicate the directions and magnitudes of the Acoustic radiation forces (ARFs). The yellow dashed lines indicate the force equilibrium positions. Radiation torque on Rayleigh PS particles in the coupled acoustic fields generated by the AFSs with e) (*θ*_1_ = 52° and *θ*_2_ = 52°) and f) (*θ*_1_ = 42° and *θ*_2_ = 52°). g) Radiation torque on a particle exerted by the coupled acoustic fields on the focal plane as a function of particle radius.

The structural parameters for AFSs with an identical initial radius and disparate initial radii are: *r*_o_ = *r*_b_ = 38.8 mm, *d*_1_ = *d*_2_ = 1.8 mm, and *r*_o_ = 27.2 mm, *r*_b_ = 38.8 mm, *d*_1_ = 1.4 mm, *d*_2_ = 1.8 mm, respectively. **Figure** **4**a,b shows the simulated intensity distributions in the focal plane after the ultrasound passes through the two AFSs. Similar to the intensity field generated in air, they both have double high‐intensity lobes. We calculated the acoustic radiative force (AFR) of Rayleigh polystyrene (PS) particles (hard particles) in coupled acoustic fields. The Rayleigh PS sphere has a diameter of 0.6 mm (0.2 *λ*), a mass density of 1050 kg m^−3^, a longitudinal wave velocity of 2170 m s^−1^ and a shear wave velocity of 1100 m s^−1^. For Rayleigh PS particles with radii much smaller than the wavelength, their AFR in the coupled acoustic field can be obtained by solving for the spatial negative gradient of the Gor'kov potential.^[^ ^49^ ^]^ The background of Figure 4c,d shows the simulated Gor'kov potential distribution of the Rayleigh PS particles in the corresponding coupled acoustic field. Comparing the acoustic intensity distribution, the Gor'kov potential is positively correlated with the acoustic intensity. The arrows in Figure 4c,d indicate the AFR suffered by the Rayleigh PS particles in the coupled acoustic fields. It can be seen that a potential valley appears between the two acoustic intensity maxima, with the direction of the AFR pointing toward the center of the acoustic tweezer field with double high‐intensity lobes. There are also two particle capture points appearing at the edges of the two acoustic intensity maxima, as shown by the yellow dashed lines in Figure 4c,d. PS particles close to the capture points can be confined inside these capture points. In contrast, PS particles initially outside the capture points will be repelled. Thus both coupled acoustic fields with different AM distributions can stably trap Rayleigh PS particles at the equilibrium positions of the potential valleys. We have also calculated the radiation torque of Rayleigh PS particles subjected to the two coupled acoustic fields. For a Rayleigh PS particle with a diameter of 0.6 mm (0.2*λ*), the radiation torque it is subjected to can be obtained by the equation $T_{z}^{spin}=\omega Im\left(\alpha_{d}\right)S_{z}$, where *α_d_* is the dipole polarization coefficient of the particle.^[^ ^41^ ^]^ Figure 4e,f shows the torque applied to this Rayleigh PS particle in the two coupled acoustic fields, the magnitude and direction of which are directly related to the spin AM. Figure 4g shows the torque versus particle radius *a* when the Rayleigh PS particle is located at the equilibrium position (center potential valley). It can be observed that when the radius of the PS particles is <0.05*λ*, the torque they are subjected to at the two equilibrium positions is essentially negligible. As the particle radius increases, particles are consistently subjected to a small torque within the coupled acoustic field comprising BAVs of identical pole angles. Conversely, the torque exhibits a pronounced surge within the coupled acoustic field comprising BAVs of disparate pole angles. When the radius of the PS particle is equal to 0.1*λ*, the radiation torque of the particle in a coupled acoustic field consisting of BAVs with the same pole angle is only 0.15 of that in the coupled acoustic field consisting of different pole angles. Thus, under the action of spin AM, the captured Rayleigh PS particles are either stationary at the equilibrium position [Figure 4e] or rotating around the central axis [Figure 4f]. In this study, the focus is on the effect of the *z* component of the spin AM. In addition, we have investigated the effect of the *x* and *y* components of the spin AM on the driving particles (not shown). The different coupled acoustic fields exhibit purely linear axial polarization at the particle capture points, so that the effects of the *x* and *y* components of the spin AM on the driving of the particles are negligible.

**Figure 5:**
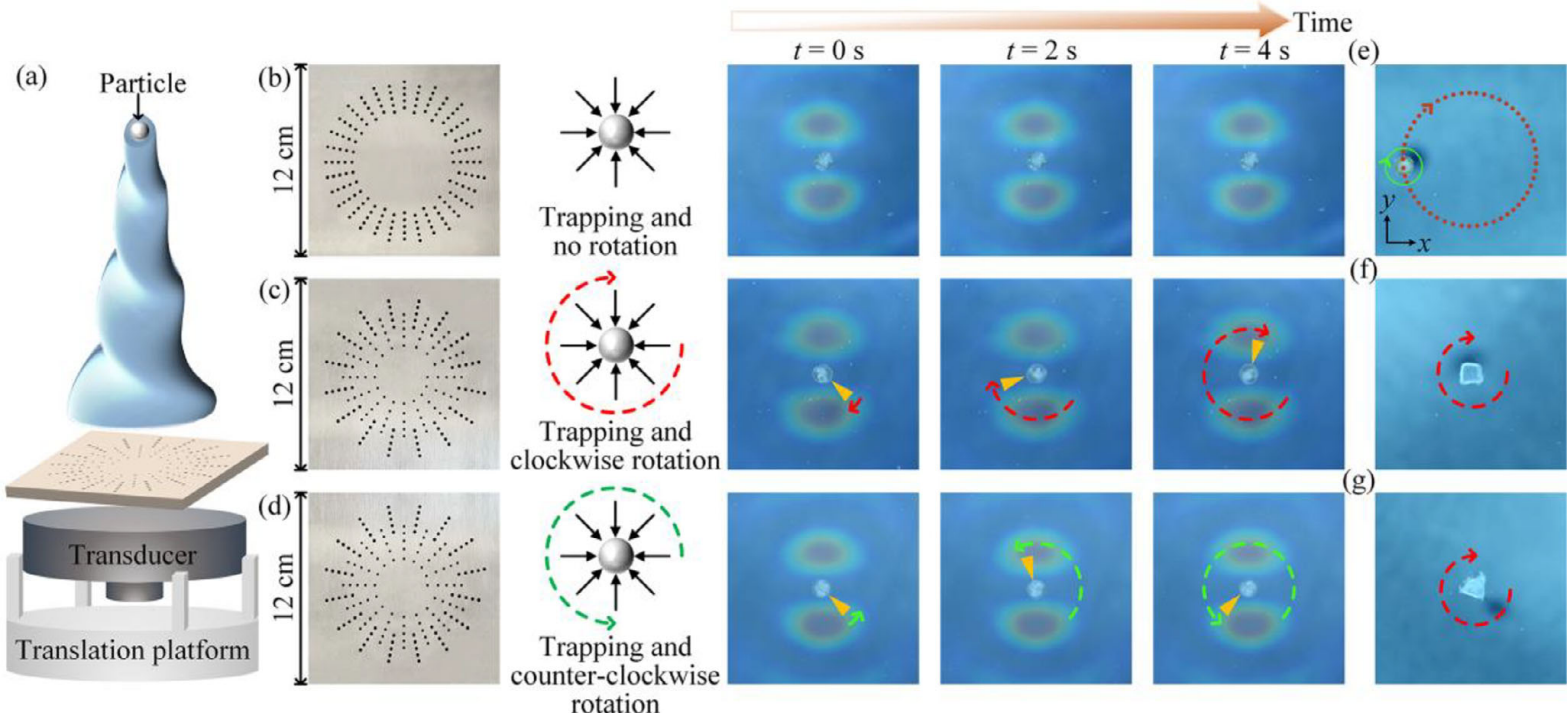
a) A schematic diagram of a device for particulate manipulation. 2D b) stationary, c) clockwise rotational, and d) counterclockwise rotational capture processes of Rayleigh particles under the action of a coupled acoustic field. On the left are ASF photographs of the corresponding processes. e) Rayleigh particles captured by rotation move along a circular path. 2D rotational capture of a f) square and g) triangular Rayleigh particle.

We further validate the theoretical predictions through particle manipulation experiments. Manipulation experiments are performed in a tank with the transducer, AFSs placed in the water, and the particles placed at the center of the water surface. The peak value of the electrical power applied to the ultrasonic transducer after the 120 mV electrical signal has passed through the high‐power amplifier is 10 W. **Figure** **5**a shows a schematic diagram of a device for particulate manipulation. The transducer is fixed to a removable platform. When the sound source is turned on, ultrasonic waves from the transducer pass through the structured plate samples, thereby producing coupled acoustic fields with different AM distributions in the water and acting on the particles. Rayleigh polypropylene particles with a diameter of 0.6 mm (0.2 *λ*) have been selected for the experimental study. These particles are hard particles and suspended on the water surface, thus facilitating the experimental observation. Acoustic waves can transfer AM to suspended particles with highly symmetric geometries, as evidenced by previous studies.^[^ ^50^ ^]^ Figure 5b shows the 2D stationary capture process of a Rayleigh particle after ultrasonic waves pass through the structured plate (see Video S1, Supporting Information). The left side of Figure 5b shows a photograph of an AFS made of stainless steel.^[^ ^43^ ^]^ When an incident plane wave is emitted on the AFS, the Rayleigh particles move rapidly toward the central potential valley and remain motionless thereafter. Figure 5c,d shows the 2D clockwise and counterclockwise rotational capture processes of Rayleigh particles after ultrasound passage through the corresponding structural plates, respectively (see Videos S2 and S3, Supporting Information). The Rayleigh particles are captured at the central potential valley position and rotated around the central axis after capture. In addition, achieving high‐resolution movement of particles after capture is necessary in the manipulation. In Figure 5e, we record the 2D motions of the Rayleigh particle when the transducer is moved through the translation stage. Since the Rayleigh particles are captured in the center of the coupled acoustic field by clockwise rotation, the Rayleigh particles follow as the transducer moves. Our integrated system successfully moved a rotationally captured Rayleigh particle along a circular path (See Video S4, Supporting Information). Figure 5f,g shows the 2D clockwise rotational capture of square and triangular Rayleigh particles achieved after ultrasound waves passed through the structured plate, respectively (see Videos S5 and S6, Supporting Information). This has potential applications in acoustically driven microrobots.

**Figure 6:**
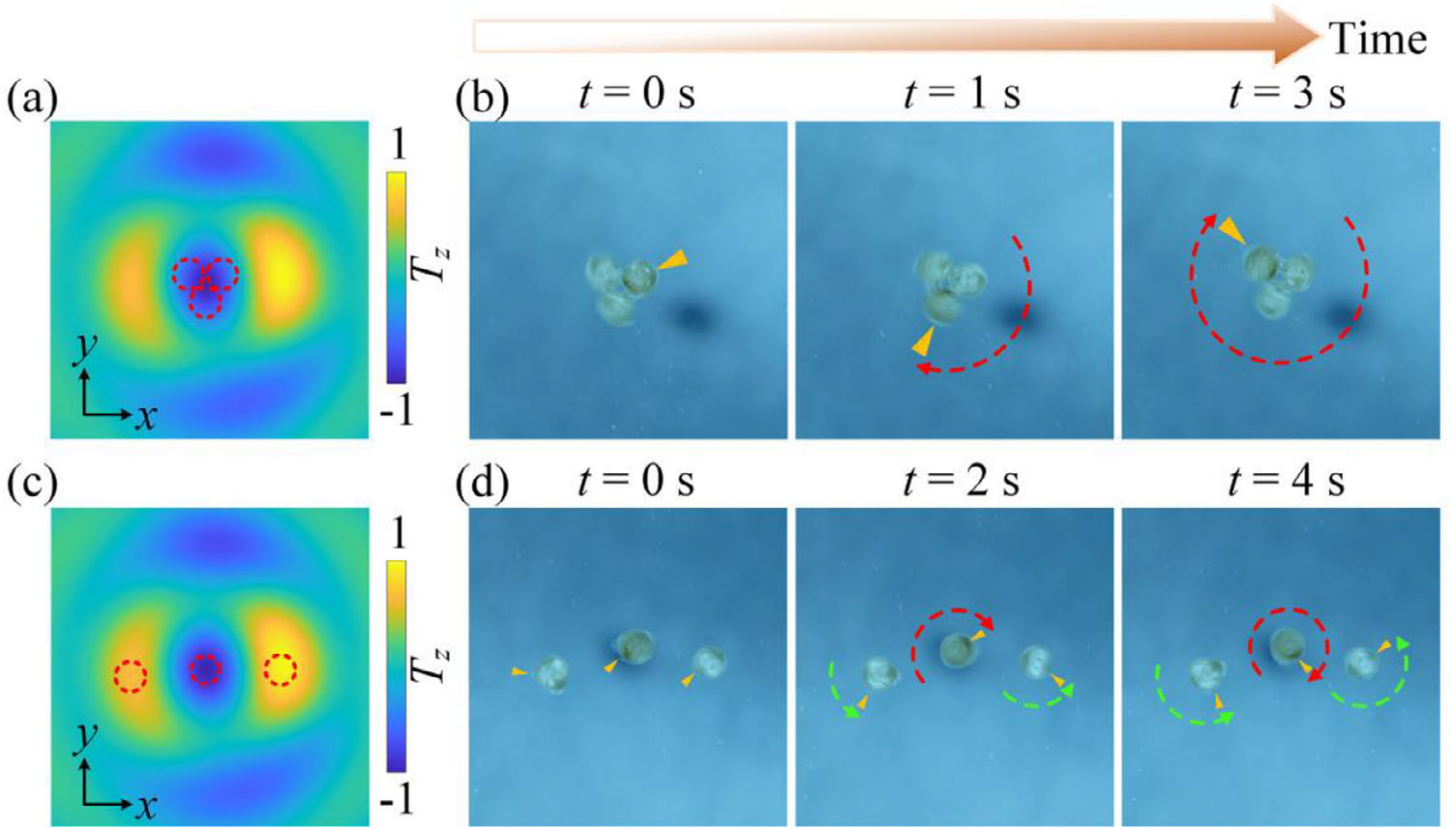
Coupled acoustic field distribution for capturing three particles a,b) at the central region and c,d) at different equilibrium positions. The red dashed circles indicate the three captured particles. The backgrounds are the torque applied to the particles in the coupled acoustic fields. States of the three captured particles (b) in the central region and (d) at three different equilibrium positions over time.

In **Figure** **6**, we show the multi‐particle manipulation achieved using a coupled acoustic field. First, we capture three particles in the central region of the coupled acoustic field. The red dashed lines in Figure 6a represent the three captured particles, and the background shows the torque they are subjected to in the coupled acoustic field, which can be used to predict the rotation of the captured particles. The series of pictures in Figure 6b depicts the clockwise rotation of this spherical structure over time (see Video S7, Supporting Information for the rotation process). Other fabricated microobjects can be envisaged to rotate in a similar manner. We further demonstrate that the coupled acoustic field can trap particles at different equilibrium positions and rotate them in the desired direction. The red dashed lines in Figure 6c represent the positions where the three captured particles are located in this case. Figure 6d shows the state of the three captured particles at different times, for the rotation of the particles over time see Video S8 (Supporting Information). Typically, the opposite rotation manipulation requires controlling the spin state of the beam. In contrast, our method only requires changing the captured positions of the particles, and thus is easier to apply in practice.

## 6 Conclusion

In this study, we investigate the conversion of acoustic spin‐orbit AM under the interaction of nonparaxial high‐order BAVs with opposite TCs. The results show that the spin and orbital AMs of the superimposed acoustic field are only related to the TC and energy density of the single‐beam waves involved in the superposition, and are independent of the energy density of the superimposed acoustic field. Spin and orbital AMs are converted as the polar angle of the superimposed BAV changes. On this basis, we have designed an AFS with two sets of circular holes to practically demonstrate the conversion of acoustic spin‐orbit AM under the interaction of BAVs. Theoretical analysis, numerical simulations, and experimental results validate the spin‐orbit AM conversion achieved by an AFS. Finally, we discuss the mechanical effects of coupled acoustic fields with different AM distributions on Rayleigh particles placed in them. Rayleigh particles with different shapes can be stationary and rotationally trapped by the coupled acoustic field. We successfully moved a rotationally captured Rayleigh particle along a circular path. In addition, we implemented multi‐particle manipulation using a coupled acoustic field. The method shows that the spin state of a particle can be controlled by changing the position of the captured particle. The different kinematic states of Rayleigh particles in coupled acoustic fields provide direct and compelling evidence for acoustic spin‐orbit AM conversion.

Our scheme provides a deeper understanding of the physical basis of the AM exhibited by acoustic vortex beams. In the future, improvements to the AFS will be made to enable dynamic transformation of spin‐orbit AM.^[^ ^45^ ^]^ Furthermore, the successful development of acoustic tweezers based on spin‐orbit AM conversion will drive a wide range of applications, such as the high‐resolution motion after particle rotation capture is highly beneficial for acoustically driven microrobots. It will provide an aid for the separation and sorting of fine biological samples, the arrangement of micro‐objects for controlled self‐assembly, and the arrangement of cellular distributions for biofabrication. To further extend the boundaries of this work, we will investigate how acoustic spin and orbit AMs can be used to manipulate biological samples such as cells, embryos, worms, and zebrafish, how they can be used to manipulate objects in flowing media, and their applications in biomedical engineering and advanced manufacturing.

## Conflict of Interest

The authors declare no conflict of interest.

## Supporting information



Supporting Information

Supplemental Video 1

Supplemental Video 2

Supplemental Video 3

Supplemental Video 4

Supplemental Video 5

Supplemental Video 6

Supplemental Video 7

Supplemental Video 8

## Data Availability

The data that support the findings of this study are available from the corresponding author upon reasonable request.
