# Supplementary material for: Spin‐Orbit Angular Momentum Conversion in Coupled Nonparaxial Bessel Acoustic Vortices
Source: Adv Sci (Weinh). 2025 Jun 10;12(33):e05631. doi: 10.1002/advs.202505631 (PMC12412625; doi:10.1002/advs.202505631)
Supplement: Supplementary file 1 — Supporting Information [file ADVS-12-e05631-s007.pdf]

ADVANCED SCIENCE
Open Access

Supporting Information

for *Adv. Sci.*, DOI 10.1002/advs.202505631

Spin-Orbit Angular Momentum Conversion in Coupled Nonparaxial Bessel Acoustic Vortices

Di-Chao Chen, Qian Mo, Da-Jian Wu, Xing-Feng Zhu, Ying Cheng and Xiao-Jun Liu**

Supporting Information

Spin-orbit angular momentum conversion in coupled nonparaxial Bessel acoustic vortices

Di-Chao Chen, Qian Mo, Da-Jian Wu, Xing-Feng Zhu, Ying Cheng, and Xiao-Jun Liu**

D. Chen, Q. Mo, D. Wu, X. Zhu
Institute of Acoustics, School of Physics and Technology, Nanjing Normal University, Nanjing 210023, China
E-mail: wudajian@njnu.edu.cn

D. Chen, X. Zhu, Y. Cheng, X. Liu
MOE Key Laboratory of Modern Acoustics, School of Physics, Nanjing University, Nanjing 210093, China
E-mail: liuxiaojun@nju.edu.cn

1. Derivation of Acoustic Orbital and Spin Angular Momentum

A nonparaxial higher Bessel acoustic vortex (BAV) pressure field can be described as

$$P = AJ_l(k_r r)\exp(il\varphi + ik_z z), \tag{S1}$$

where A is the constant amplitudes of the acoustic vortex, l is the topological charge (TC) of the vortex, $J_l(x)$ is the lth-order first kind Bessel function, $k_z = k\cos\theta$ and $k_r = k\sin\theta$ are the axial and radial wave numbers of the vortex, respectively, θ is the polar angle of the Bessel beam, and (r, φ, z) is the cylindrical coordinate in real space. The complex conjugate form of the acoustic pressure field is $P^* = AJ_l(k_r r)\exp(-il\varphi - ik_z z)$. Substituting the acoustic pressure field into $\nabla P = i\rho\omega\vec{v}$ yields the components of the velocity

$$\begin{cases} v_r = \dfrac{A}{\rho\omega}\dfrac{d\,J_l(k_r r)}{dr}\exp(il\varphi + ik_z z) \\ \quad = -i\dfrac{Ak_r}{2\rho\omega}\left[J_{l-1}(k_r r) - J_{l+1}(k_r r)\right]\exp(il\varphi + ik_z z), \\ v_\varphi = \dfrac{A}{\rho\omega}\dfrac{lJ_l(k_r r)}{r}\exp(il\varphi + ik_z z) \\ \quad = \dfrac{Ak_r}{2\rho\omega}\left[J_{l-1}(k_r r) + J_{l+1}(k_r r)\right]\exp(il\varphi + ik_z z), \\ v_z = \dfrac{Ak_z}{\rho\omega}J_l(k_r r)\exp(il\varphi + ik_z z). \end{cases} \tag{S2}$$

and

$$\begin{cases} v_r^{\,*} = -i\dfrac{Ak_r}{2\rho\omega}\left[J_{l-1}(k_r r) - J_{l+1}(k_r r)\right]\exp(-il\varphi - ik_z z), \\ v_\varphi^{\,*} = \dfrac{Ak_r}{2\rho\omega}\left[J_{l-1}(k_r r) + J_{l+1}(k_r r)\right]\exp(-il\varphi - ik_z z), \\ v_z^{\,*} = \dfrac{Ak_z}{\rho\omega}J_l(k_r r)\exp(-il\varphi - ik_z z). \end{cases} \tag{S3}$$

$v_r^{\,*}$, $v_\varphi^{\,*}$, and $v_z^{\,*}$ stands for the complex conjugate of the velocity. Substituting the vector velocity part of the acoustic field into $W = \frac{1}{4}\beta P \cdot P^* + \frac{1}{4}\rho(v_r \cdot v_r^{\,*} + v_\varphi \cdot v_\varphi^{\,*} + v_z \cdot v_z^{\,*})$ and using the dispersion relation $\omega^2 = k^2c^2 \equiv \frac{k^2}{\rho\beta}$, the energy density of an individual BAV can be obtained

$$W = \frac{\beta A^2}{8}\left\{2(1+\cos^2\theta_0)J_l^2(k_r r) + \sin^2\theta_0\left[J_{l-1}^2(k_r r) + J_{l+1}^2(k_r r)\right]\right\}. \tag{S4}$$

Substituting the vector velocity part of the acoustic field into $\vec{S} = \frac{\rho}{2\omega}\mathrm{Im}(\vec{v}^{\,*} \times \vec{v})$, the spin AM of an individual BAV can be obtained

$$S_z = \frac{\beta A^2}{4\omega}\sin^2\theta_0\left[J_{l-1}^2(k_r r) - J_{l+1}^2(k_r r)\right]. \tag{S5}$$

Based on the canonical momentum density $\vec{p} = \frac{\vec{\Pi}}{c^2} - \frac{1}{4}\nabla \times \vec{S}$ and the energy flux density

$\vec{\Pi} = \frac{1}{2}\mathrm{Re}(P^*\vec{v})$, the orbital AM of a BAV can be computed from $\vec{L} = \vec{r} \times \vec{p}$ as

$$\begin{aligned} L_z = {} & \frac{\beta A^2 l}{8\omega}\left\{2(1+\cos^2\theta_0)J_l^2(k_r r) + \sin^2\theta_0\left[J_{l-1}^2(k_r r) + J_{l+1}^2(k_r r)\right]\right\} \\ & - \frac{\beta A^2}{8\omega}\left[J_{l-1}^2(k_r r) - J_{l+1}^2(k_r r)\right]. \end{aligned} \tag{S6}$$

The acoustic pressure field of two BAVs with opposite TCs superimposed can be described as

$$P(s) = P_1 + P_2 = AJ_{l_1}(k_{r_1}r)\exp(il_1\varphi + ik_{z_1}z) + BJ_{l_2}(k_{r_2}r)\exp(il_2\varphi + ik_{z_2}z). \tag{S7}$$

The components of the velocity field can be obtained from the superimposed acoustic field P(s) as

$$\begin{cases} v_r(\mathrm{s}) = -i\frac{Ak_{r_1}}{2\rho\omega}\left[J_{l_1-1}(k_{r_1}r) - J_{l_1+1}(k_{r_1}r)\right]\exp(il_1\varphi + ik_{z_1}z) \\ \qquad -i\frac{Bk_{r_2}}{2\rho\omega}\left[J_{l_2-1}(k_{r_2}r) - J_{l_2+1}(k_{r_2}r)\right]\exp(il_2\varphi + ik_{z_2}z), \\ v_\varphi(\mathrm{s}) = \frac{Ak_{r_1}}{2\rho\omega}\left[J_{l_1-1}(k_{r_1}r) + J_{l_1+1}(k_{r_1}r)\right]\exp(il_1\varphi + ik_{z_1}z) \\ \qquad + \frac{Bk_{r_2}}{2\rho\omega}\left[J_{l_2-1}(k_{r_2}r) + J_{l_2+1}(k_{r_2}r)\right]\exp(il_2\varphi + ik_{z_2}z), \\ v_z(\mathrm{s}) = \frac{Ak_{z_1}}{\rho\omega}J_{l_1}(k_{r_1}r)\exp(il_1\varphi + ik_{z_1}z) + \frac{Bk_{z_2}}{\rho\omega}J_{l_2}(k_{r_2}r)\exp(il_2\varphi + ik_{z_2}z). \end{cases} \tag{S8}$$

The energy density obtained as

$$\begin{aligned} W(\mathrm{s}) = {} & \frac{\beta A^2}{8}\left\{2(1+\cos^2\theta_1)J_{l_1}^2(k_{r_1}r) + \sin^2\theta_1\left[J_{l_1\text{-}1}^2(k_{r_1}r) + J_{l_1+1}^2(k_{r_1}r)\right]\right\} \\ & + \frac{\beta B^2}{8}\left\{2(1+\cos^2\theta_2)J_{l_2}^2(k_{r_2}r) + \sin^2\theta_2\left[J_{l_2\text{-}1}^2(k_{r_2}r) + J_{l_2+1}^2(k_{r_2}r)\right]\right\} \\ & + \frac{\beta AB}{8}\left\{2(1+\cos\theta_1\cos\theta_2)J_{l_1}(k_{r_1}r)J_{l_2}(k_{r_2}r) + \sin\theta_1\sin\theta_2\left[J_{l_1\text{-}1}(k_{r_1}r)J_{l_2\text{-}1}(k_{r_2}r)\right.\right. \\ & \left.\left. + J_{l_1+1}(k_{r_1}r)J_{l_2+1}(k_{r_2}r)\right]\right\}\left\{\exp\left[i(l_1-l_2)\varphi + i(k_{z_1}-k_{z_2})z\right]\right. \\ & \left. + \exp\left[-i(l_1-l_2)\varphi - i(k_{z_1}-k_{z_2})z\right]\right\}. \end{aligned} \tag{S9}$$

In the context of two BAVs with opposite TCs superimposed on each other, the conjugate multiplication of P and v does not result in the elimination of the e exponent term.

Consequently, W is complex. The axial spin AM density obtained as

$$
\begin{aligned}
S_z(\mathrm{s}) = {} & \frac{\beta}{4\omega}\left\{A^2\sin^2\theta_1\left[J_{l_1-1}^2(k_{r_1}r)-J_{l_1+1}^2(k_{r_1}r)\right]+B^2\sin^2\theta_2\left[J_{l_2-1}^2(k_{r_2}r)-J_{l_2+1}^2(k_{r_2}r)\right]\right\} \\
& +\frac{\beta AB}{k^2r^2\omega}\cos\left[(l_1-l_2)\varphi+(k_{z_1}-k_{z_2})z\right]\left[krl_2\sin\theta_1 J_{l_1-1}(k_{r_1}r)J_{l_2}(k_{r_2}r)\right. \\
& \left. -2l_1l_2J_{l_1}(k_{r_1}r)J_{l_2}(k_{r_2}r)+krl_1\sin\theta_2 J_{l_1}(k_{r_1}r)J_{l_2-1}(k_{r_2}r)\right],
\end{aligned}
\tag{S10}
$$

The axial orbital AM density obtained as

$$
\begin{aligned}
L_z(\mathrm{s}) = {} & \frac{\beta}{16k^2r^2\omega}\Big\langle A^2k^2r^2l_1J_{l_1}^2(k_{r_1}r)(7+\cos 2\theta_1)+B^2k^2r^2l_2J_{l_2}^2(k_{r_2}r)(7+\cos 2\theta_2) \\
& +AB\cos\left[(l_1-l_2)\varphi+(k_{z_1}-k_{z_2})z\right]J_{l_1}(k_{r_1}r)\Big\{\Big[4k^2r^2(l_1+l_2)(1+\cos\theta_1\cos\theta_2) \\
& +16l_1l_2+8l_1l_2^{\,2}+2k^2r^2l_2\sin^2\theta_1\Big]J_{l_2}(k_{r_2}r)-4krl_1(2+l_2)\sin\theta_2J_{l_2-1}(k_{r_2}r)\Big\} \\
& +kr\Big\{A^2k^2r^2\sin^3\theta_1\left[J_{l_1-1}(k_{r_1}r)J_{l_1-2}(k_{r_1}r)+J_{l_1+1}(k_{r_1}r)J_{l_1+2}(k_{r_1}r)\right] \\
& +B^2k^2r^2\sin^3\theta_2\left[J_{l_2-1}(k_{r_2}r)J_{l_2-2}(k_{r_2}r)+J_{l_2+1}(k_{r_2}r)J_{l_2+2}(k_{r_2}r)\right] \\
& +2ABl_2\cos\left[(l_1-l_2)\varphi+(k_{z_1}-k_{z_2})z\right]\sin\theta_1J_{l_2}(k_{r_2}r)\Big[2l_1J_{l_1+1}(k_{r_1}r) \\
& -2\left(1+l_1+l_2\right)J_{l_1-1}(k_{l_1}r)+kr\sin\theta_1J_{l_1-2}(k_{r_1}r)\Big]+2ABkr\Big[\left(l_1+2l_2\right)J_{l_1-1}(k_{r_1}r) \\
& -l_1J_{l_1+1}(k_{r_1}r)\Big]\cos\left[(l_1-l_2)\varphi+(k_{z_1}-k_{z_2})z\right]\sin\theta_1\sin\theta_2J_{l_2-1}(k_{r_2}r)\Big\}\Big\rangle,
\end{aligned}
\tag{S11}
$$

Setting A = B = 1 and the TCs of the two BAVs are $l_1 = -l_2$. Summing the spin AM and orbital AM of the superimposed acoustic field and simplifying gives

$$
L_z(\mathrm{s})+\frac{S_z(\mathrm{s})}{2}=\frac{(l_1W_1-l_1W_2)}{\omega}, \tag{S12}
$$

where W_1 and W_2 are the energy densities of individual BAVs. To further validate the formula, we show the AM distribution of two BAVs superimposed with amplitude A = B = 1, topological charges l_1 = 1 and l_2 = −1, and polar angles θ_1 = 30° and θ_2 = 60°, as shown in Figure S1(a). The AM distribution obtained according to $(l_1W_1 + l_2W_2)/\omega$ is shown in Figure S1(b). In addition, the AM distributions of two BAVs superimposed with amplitude A = B = 1, topological charges l_1 = 2 and l_2 = −2, and polar anglesθ_1 = 30° and θ_2 = 60°, and the AM distribution obtained by $(l_1W_1 + l_2W_2)/\omega$ are shown in Figures S1 (c) and S1 (d), respectively. We can observe that $L_z+S_z/2$ after the superposition of two BAVs has the same distribution shape as $(l_1W_1 + l_2W_2)/\omega$, which further verifies the correctness of equation (S12).

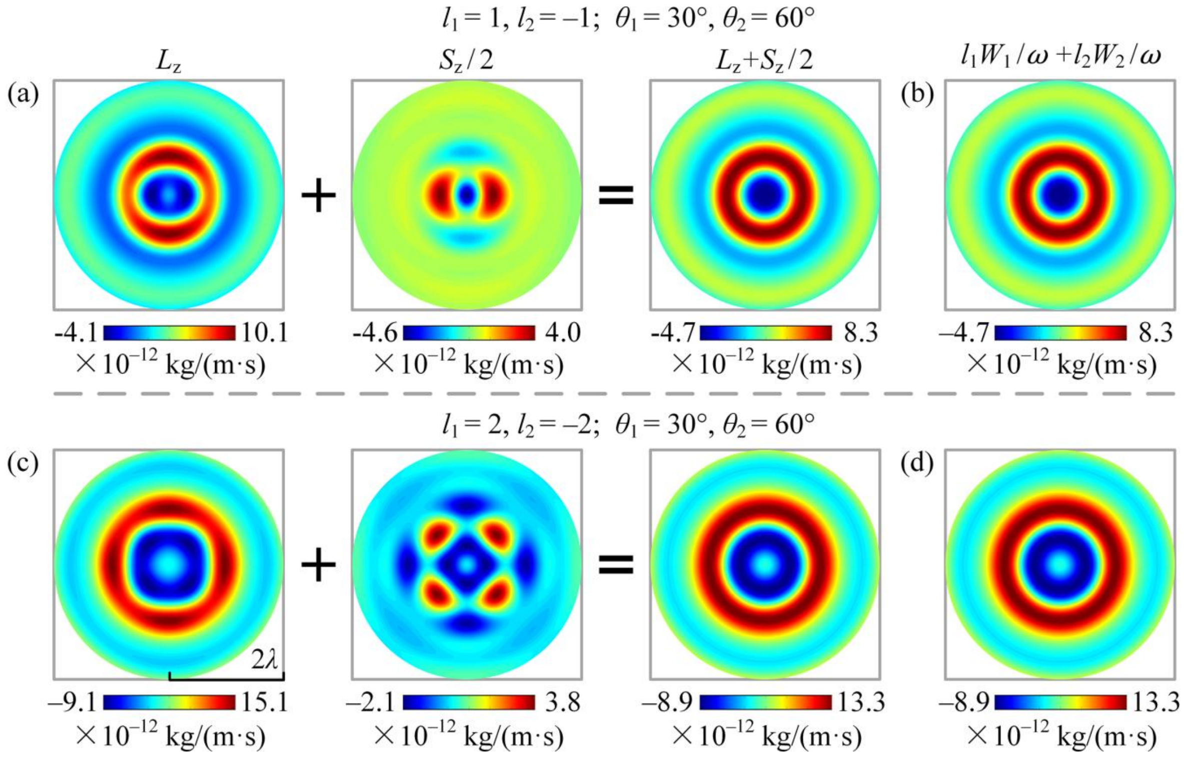

Figure S1. A superposition of two BAVs with TCs l_1 = 1 and l_2 = −1, and polar angles θ_1 = 30 °and θ_2 = 60 °. Corresponding AM distribution obtained according to (a) L_z+S_z/2 and (a) (l_1W_1+ l_2W_2)/ω. A superposition of two BAVs with TCs l_1 = 2 and l_2 = −2, and polar angles θ_1 = 30 ° and θ_2 = 60 °. Corresponding AM distribution obtained according to (c) L_z+S_z/2 and (d) (l_1W_1+ l_2W_2)/ω.

2. Influence of observation distance on Spin-orbit AM conversion

We further investigated the distributions of acoustic intensity, phase and spin AM density near the focusing plane. Figure S2(a) shows the distribution of acoustic intensity in the *x*-*z* plane obtained from the simulation. It can be seen that there are two finite-length foci along the *z*-direction, which indicate the convergence region of the acoustic energy. Figures S2 (b), S2(c) and S2(d) show the distribution of acoustic intensity, phase and spin AM density in the three transverse planes at $z = 9\lambda$, $z = 10\lambda$ and $z = 11\lambda$, respectively. As z increases to 11λ or decreases to 9λ, the corresponding acoustic intensity decreases. In addition the acoustic intensity distribution undergoes a rotation, which is an acoustic spin-controlled orbital rotation, as we have demonstrated in previous studies [s1]. The phase and spin AM density distributions are essentially the same as those at the focal plane. These results demonstrate the acoustic spin-orbit AM conversion that is also achievable by the proposed AFS near the focusing plane.

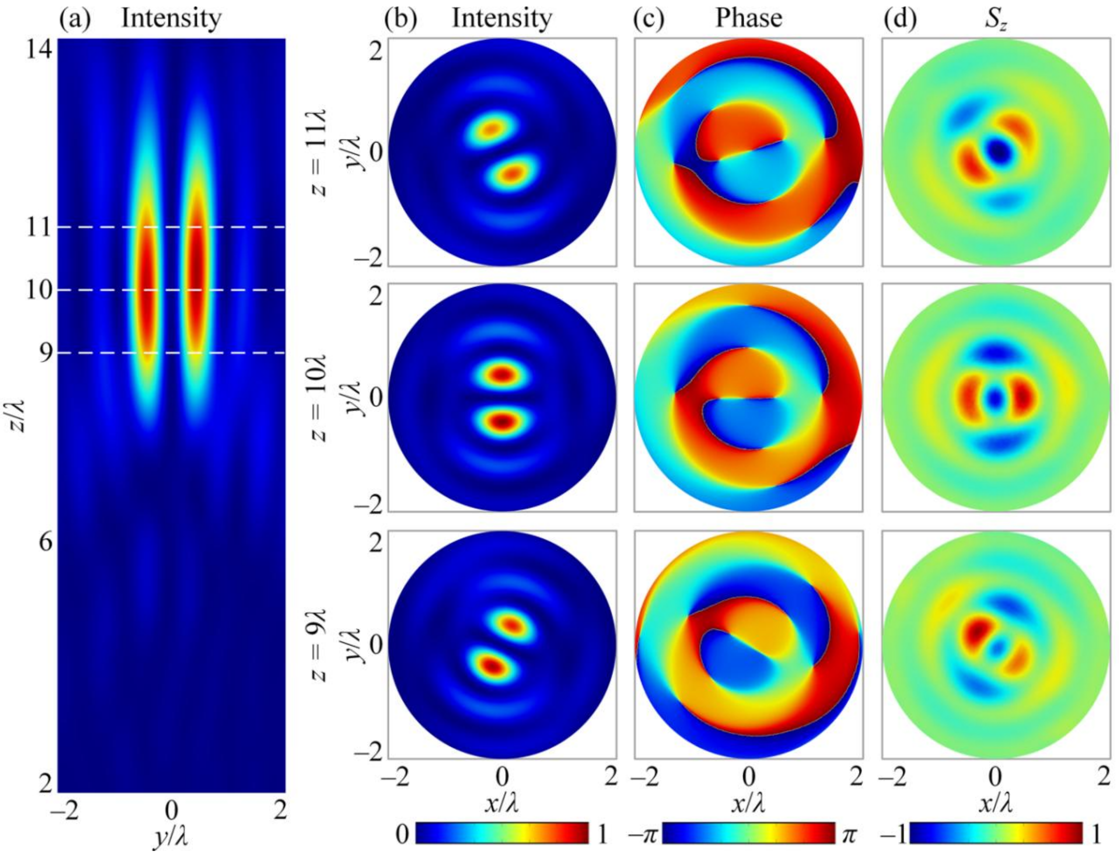

Figure S2. (a) Simulated coupled acoustic intensity distribution in the *x*-*z* plane. (b) Simulated acoustic intensity, (c) phase, and (d) spin AM density distributions at three transverse planes of $z = 9\lambda$, $z = 10\lambda$ and $z = 11\lambda$.

3. Influence of frequency on Spin-orbit AM conversion

To further investigate the frequency response characteristics of the spin-orbit AM conversion, we present the simulation results of the AFS for the realized spin-orbit angular momentum conversion at different frequencies, as shown in Fig. S3. The operating frequency fluctuates by 12%, i.e., the test range is from 13.2 to 16.8 kHz. Focus intensity contrast and S_z contrast in the central region of coupled acoustic field as a function of frequency are shown in Figures S3(a) and S3(b), respectively. It is observed that when the frequency is increased to 16.5 kHz or decreased to 13.6 kHz, the acoustic intensity decreases to half of the acoustic intensity at the set frequency. Due to the change in frequency, the acoustic intensity changed relative to the position at the focal plane. Figure S3(c) show the simulated coupled acoustic intensity, phase, and spin AM density distributions at different working frequencies. The results show that spin-orbit AM conversion is still well achieved when the operating frequency fluctuates between 8% (i.e., 13.8 ~ 16.2 kHz).

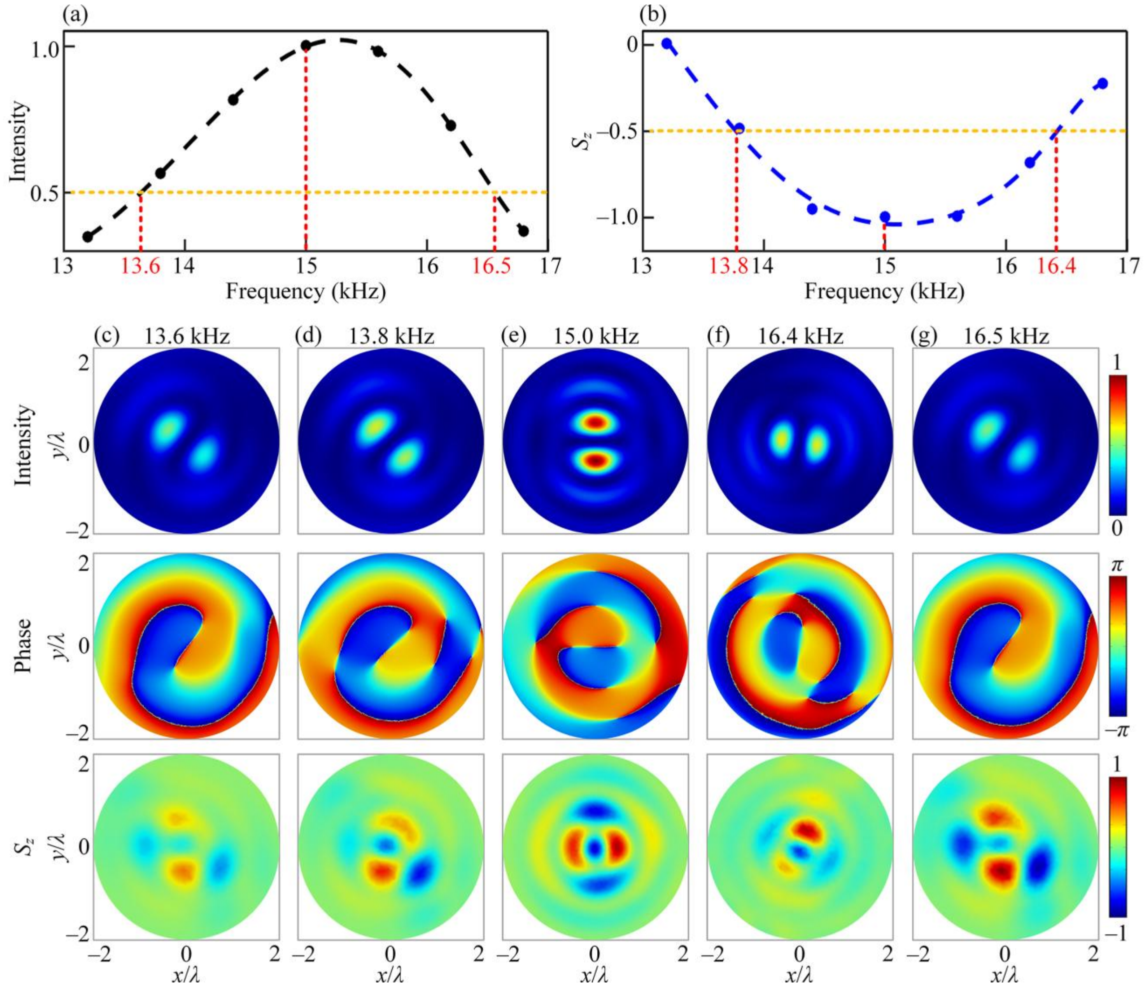

Figure S3. (a) Focus intensity contrast and (b) S_z contrast in the central region of coupled acoustic field as a function of frequency. (c) Simulated coupled acoustic intensity, phase, and spin AM density distributions at different working frequencies.

References

[S1] D. Chen, X. Liu, D. Wu, X. Zhu, Q. Wei, Y. Cheng, X. Liu, Commun. Phys. **2024**, 7, 212.
